# Under what conditions? Therapist and client characteristics moderate the role of change talk in brief motivational intervention

**DOI:** 10.1186/1940-0640-8-S1-A29

**Published:** 2013-09-04

**Authors:** Jacques Gaume, Molly Magill, Richard Longabaugh, Nicolas Bertholet, Gerhard Gmel, Jean-Bernard Daeppen

**Affiliations:** 1 Center for Alcohol and Addiction Studies, Brown University, Providence, RI, USA; 2 Alcohol Treatment Centre, Lausanne University Hospital, Lausanne, Switzerland

## 

Client change talk has been proposed as a mechanism of change in motivational interviewing (MI) by mediating the link between therapist MI-consistent behaviors (MICO) and client behavioral outcomes. In the present study, we tested under what circumstances this generic MI conceptualization was supported. The study context was a Swiss clinical trial of brief MI for heavy drinking among non-treatment seeking young men (age 20). We conducted psycholinguistic coding of 174 sessions using the MI Skill Code (MISC 2.1, Miller et al. 2008) and derived the frequency of MICO and the strength of change talk (CTS) averaged over the session. CTS was examined as a mediator of the relationship between MICO and a drinking composite score (including drinking days per week, drinks per drinking day, and binge drinking frequency) measured 3 months after baseline, controlling for the baseline drinking composite score. Finally, we tested therapist gender and MI experience and client readiness to change and alcohol problems severity as moderators of this mediation model (see Hayes 2013). CTS significantly predicted outcome in the expected direction (i.e. higher strength related to less drinking), but MICO did not significantly predicted CTS. However, CTS mediated the relationship between MICO and drinking outcomes when therapists had more experience in MI and when clients had more severe alcohol problems (i.e. significant conditional indirect effects). Findings showed that the mechanism of change hypothesized by MI theory was operative in our brief MI model (mean=19, SD=5 minutes) with heavy drinking young men, but only under particular conditions. Our results suggest that particular attention should be paid to therapist selection and/or training and supervision until they reach a certain level of competence, and that MI might not be appropriate for non-treatment seeking clients drinking at lower level of risk.

